# Admission patterns in a psychiatric intensive care unit in Ireland: a longitudinal follow-up

**DOI:** 10.1192/bjo.2021.750

**Published:** 2021-06-18

**Authors:** Shaeraine Raaj, Sujesha Navanathan, Basil Matti, Anisha Bhagawan, Pauline Twomey, John Lally, Roy Browne

**Affiliations:** 1 Mater Misericirdiae University Hospital, Phoenix Care Centre; 2 National Forensic Mental Health Services; 3 St. Patrick`s University Hospital; 4 national drug treatment centre; 5 Phoenix Care Centre; 6 Mater Misericirdiae University Hospital

## Abstract

**Aims:**

This observational study aims to describe the course of the admission and clinical characteristics of admissions to the PICUs in the Phoenix Care Centre, Dublin, Ireland. The authors hypothesised that the length of stay (LOS) would be shorter in male patients as compared to females.

**Method:**

This retrospective cohort study was carried out at the Phoenix Care Centre Dublin, Ireland. Informed consent was not sought as this was a retrospective chart study involving anonymised clinical data which was collected as part of routine clinical care and no items of information were reported that would enable the identification of any subject. We described primary outcomes using frequencies, percentages, mean and standard deviations, median and interquartile ranges (IQR). Between groups comparisons were made using x2 tests for categorical variables; t-tests, ANOVA tests, or Kruskal-Wallis tests, for continuous variables; All analyses were two-tailed, and a P-value ≤ 0.05 was considered statistically significant

**Result:**

Over the study period from Jan 2014 to Jan 2017 inclusive, there were 96 admission episodes to the PICU. The mean age of admitted cases was 37.1 (SD = 11.3) years (range 18–63 years). The mean length of stay (LOS) was 59.3 (SD = 61.0) days (median 39.5 days). All patients were admitted under the Mental Health Act legislation. We identified assault as the primary risk factor for pre-admission 62% (n = 62) to the PICU. Antipsychotic polypharmacy was used in 61% (n = 55) of the admission. The mean daily antipsychotic dosage was 139.4 % (SD = 65.1) of BNF maximum daily dose. A diagnosis of acute psychotic disorder (B= -1.027, p = 0.003, 95% CI: –1.691 to –0.363) was associated with reduced LOS in PICU. Majority of admissions 43% (n = 39) had a diagnosis of schizophrenia, followed by Bipolar affective disorder BPAD 21% (n = 21), schizoaffective disorder 18% (n = 18), and acute psychotic disorder 9% (n = 9).

**Conclusion:**

Psychiatric Intensive Care Unit is an essential service for the severely ill psychiatric patients and is a progressively developing sub-speciality. An important finding from our study describes the cohort of patients admitted being predominantly male, younger-aged, single, with a diagnosis of schizophrenia, legally detained, and from an Irish background. The primary indication for a referral is the risk of assault, showing the need for the intensive and secure treatment model that a PICU can provide.

